# Value of Clinical Tests in Diagnosing Anterior Cruciate Ligament Tears: What Is New?

**DOI:** 10.1055/s-0045-1813005

**Published:** 2025-12-10

**Authors:** Long Thanh Nguyen, Son Van Truong, Uyen Thi Phuong Nguyen

**Affiliations:** 1 Department of Surgery, Danang Hospital, Danang, Vietnam; 2 Department of Radiology, Vinhduc Hospital, Quangnam, Vietnam

**Keywords:** anterior cruciate ligament injuries, knee injuries, meniscus, rupture, lesões do ligamento cruzado anterior, lesões do joelho, menisco, ruptura

## Abstract

**Objective:**

To investigate the value of clinical diagnostic tests for diagnosing anterior cruciate ligament (ACL) tears with the anterior drawer, Lachman, Pivot-shift, lever sign, and Forced Active Buckling (FAB)-sign tests.

**Methods:**

A cross-sectional study was conducted on 165 knee injury patients who were indicated for knee arthroscopy from January to December 2022. The clinical examination results were compared with the procedure's gold standard to determine the value of clinical diagnostic tests.

**Results:**

The sensitivity and specificity values, respectively, were anterior drawer test: 77.5% and 86.1%; Lachman: 87.6% and 88.9%; Pivot-shift: 65.9% and 94.4%; Lever sign: 93.8% and 94.4%; and FAB sign: 81.4% and 97.2%.

**Conclusion:**

There are various clinical diagnostic tests for ACL tears. The Lever sign is a useful clinical test for physicians to examine and diagnose this condition.

## Resumo

**Objetivo:**

Investigar o valor dos exames clínicos para diagnóstico de rupturas do ligamento cruzado anterior (LCA) com os testes da gaveta anterior, Lachman, *pivot-shift* , sinal de alavanca e sinal de flambagem ativa forçada (FAB, do inglês *forced active buckling* ).

**Métodos:**

Este estudo transversal foi conduzido com 165 pacientes com lesão no joelho e indicação para artroscopia, entre janeiro e dezembro de 2022. Os resultados dos exames clínicos foram comparados ao padrão-ouro do procedimento para determinar o valor dos testes diagnósticos.

**Resultados:**

Os valores de sensibilidade e especificidade foram, respectivamente: teste da gaveta anterior: 77,5% e 86,1%; Lachman: 87,6% e 88,9%; *pivot-shift* : 65,9% e 94,4%; sinal de alavanca: 93,8% e 94,4%; e sinal de FAB: 81,4% e 97,2%.

**Conclusão:**

Há diversos exames clínicos para rupturas do LCA. O sinal da alavanca é um teste clínico útil para exame e diagnóstico dessa condição.

## Introduction

The anterior cruciate ligament (ACL) plays a pivotal role in stabilizing the knee joint by preventing excessive forward movement and internal tibia rotation. An ACL tear, whether partial or complete, constitutes a substantial knee injury. The annual incidence rate within the general population ranges from 30 to 80 cases per 100,000 person-years. This injury is notably prevalent among younger individuals, with approximately 70% of cases stemming from sports-related trauma. 1 2

Early diagnosis of ACL injuries is crucial for selecting the appropriate treatment regimen and optimizing outcomes. 3 The gold standard for this condition is direct arthroscopic imaging, though magnetic resonance imaging (MRI) findings are also considered a reference standard, with sensitivity and specificity ranging from 94 to 98%. 4 However, not every healthcare facility in less developed countries has access to the necessary diagnostic equipment. Therefore, clinical examination for detecting ACL injuries remains highly valuable.

The three most widely accepted clinical methods for diagnosing ACL tears include the anterior drawer, Lachman, and pivot-shift tests. In 2014, Italian physician Allesandro Lelli published a research report on the lever sign test (also known as the Lelli test), conducted on 400 patients. Compared with MRI, preliminary results showed nearly 100% sensitivity in diagnosing partial or complete ACL tears, both acute and chronic. This is a clinically feasible method that is not dependent on the acuteness of the injury. 3

In 2020, Fabian Blanke, a German orthopedic surgeon, introduced a new clinical method for diagnosing ACL tears in the subacute phase called the Forced Active Buckling (FAB) sign. This method is believed to have higher sensitivity and specificity than the Lachman and pivot-shift tests. 5 To enhance the diagnostic arsenal for ACL tears in clinical practice, the present research was conducted to investigate the value of various clinical methods in diagnosing ACL injuries.

## Materials and Methods

### Participants

This was a cross-sectional study conducted on 165 knee injury patients who were indicated for knee arthroscopy surgery at Danang Hospital from January to December 2022. The inclusion criteria were as follows: individuals (1) who are 14-years or older, (2) with history of knee injury, and (3) having a scheduled unilateral arthroscopic surgery. Individuals were excluded from the study if they had a history of knee surgery, knee infection, bilateral knee diseases, or fractures around the knee.

All participants provided written informed consent. The study was conducted in accordance with the Declaration of Helsinki and approved by the review board under the number HDYD2021/45, on December 15, 2021.

### Study Design

Patients were selected for the study from the daily surgery schedule. The general information recorded was age, gender, knee injury, cause of injury, stages of injury (acute < 3 weeks, subacute 3–12 weeks, chronic ≥ 12 weeks). Then, we evaluated clinical preoperative examinations by only one orthopedic surgeon (without knowing the patient's diagnosis).

Knee arthroscopies were performed by our senior surgeon, who specialized in knee surgery and was not aware of physical examination results. The intraoperative results (whether the ACL is torn or not) were documented. Then, we compared the clinical examination results with the gold standard to determine the value of clinical tests.

### Physical Examinations

Anterior drawer, Lachman, and pivot-shift tests were conducted, as described in the literature. 2

For the lever sign test (or Lelli), the patient was laid in a supine position on a rigid surface with the knee fully extended. The examiner placed one hand under the lower third of the calf and exerts a moderate downward force on the lower third of the quadriceps with the other hand. A positive test is indicated when the heel does not lift off the examination table. This suggests an ACL tear, as the tibia fails to lever forward with the applied force on the femur. Conversely, a negative test occurs when the heel lifts off the table. 6

In the FAB-sign test (describing examination of the left leg), the patient was placed in a supine position. The examiner stood at their left side, flexing the left hip until the knee is around 20 to 30° of flexion. The left hand grasped the patient's ankle and applied an internal rotation to the leg, while the right hand was placed over the knee, exerting a slight external torsional force. The patient was then instructed to actively extend the knee. The test is considered positive if there is a distinct anterior tibial subluxation, indicating an ACL tear. A negative test occurs if the patient can fully extend the knee without tibial subluxation. 5

### Statistical Analysis

The IBM SPSS Statistics for Windows (IBM Corp.), version 20.0, was used for statistical analysis. Sensitivity, specificity, positive predictive value (PPV), negative predictive value (NPV), and accuracy were used to describe diagnostic performance.

## Results

The age of 165 patients averaged 37.3 ± 12.4 years (17–65 years). A total of 107 male and 58 female patients, with 90 left and 75 right involved knees were included in the study. The primary causes of injury were traffic accidents (41.3%) and sports injuries (44.2%), as shown in Table 1 .

**Table 1:** Basic demographic and epidemiologic data

Variables	Number (%)
**Age, years (mean ± SD)**	37.3 ± 12.4 [17;65]
**Gender (male/female)**	107(64.5) / 58 (35.2)
**Side (left/right)**	90 (54.5) / 75 (45.5)
**Causes of injury**	
Traffic accident	68 (41.3)
Sports injuries	73 (44.2)
Personal accident	18 (10.9)
Work accident	6 (3.6)
**Stages of injury**	
Acute	54 (32.7)
Subacute	61 (37.0)
Chronic	50 (30.3)
**Arthroscopic diagnosis (ACL injury yes/no)**	129 (78.2) / 36 (21.8)

**Abbreviations:** ACL, anterior cruciate ligament; SD, standard deviation.

Clinical tests with high sensitivity in diagnosing ACL tears are the Lever sign (93.8%), Lachman (87.6%), and FAB-sign (81.4%). The ones with highest specificity are FAB-sign (97.2%), Lever sign (94.4%), and Pivot-shift (94.4%). All five clinical tests have a high positive predictive value of over 90% ( Table 2 ).

**Table 2:** The diagnostic values of the five tests detecting an ACL tears

Items	ADT	LT	PST	LST	FABST
True-positive, n	100	113	85	121	105
True-negative, n	31	32	34	34	35
False-positive, n	5	4	2	2	1
False-negative, n	29	16	44	8	24
Sensitivity, %	77.5	87.6	65.9	93.8	81.4
Specificity, %	86.1	88.9	94.4	94.4	97.2
Accuracy, %	79.4	87.9	72.1	93.9	84.8
PPV, %	95.2	96.6	97.7	98.4	99.1
NPV, %	51.7	66.7	43.6	81.0	59.3

**Abbreviations:** ACL, anterior cruciate ligament; ADT, Anterior Drawer Test; FABST, Forced Active Buckling Sign Test; LST, Lever Sign Test; LT, Lachman Test; NPV, negative predictive value; PPV, positive predictive value; PST, Pivot-Shift Test.

## Discussion

The ACL is a crucial structure for maintaining knee joint stability, making early diagnosis of such injuries essential for effective treatment and prevention of further damage. Traditionally, clinical diagnosis of ACL tears relies on three tests: anterior drawer, Lachman, and pivot-shift.

Historically, the anterior drawer test has been considered the most popular and widely used method. However, it lacks the sensitivity required to diagnose acute ACL tears compared with chronic cases. The Lachman test is regarded as the most accurate and reliable method for diagnosis, while the Pivot-shift one is considered the most specific but least sensitive of the three. 7 8

In our study, we observed the sensitivity and specificity of the anterior drawer test to be 77.5% and 86.1%, the Lachman's to be 87.6% and 88.9%, and the Pivot-shift's to be 65.9% and 94.4%. In the most recent meta-analysis by Huang et al., 9 the results for the sensitivity and specificity of the anterior drawer test were 64% and 87%, the Lachman test's was 76% and 89%, and the Pivot-shift's was 59% and 97%. Sokal et al. 10 found that the sensitivity and specificity of the anterior drawer test were 83% and 85%, the Lachman's was 81% and 85%, and the Pivot-shift's was 55% and 94%.

Our results regarding classic tests are consistent with the literature. These studies all indicate that the accuracy of classic tests is influenced by various factors, such as swelling, reactive joint inflammation, and muscle guarding due to pain. 11 Furthermore, a small hand size of the examiner or a large foot size of the patient can make it difficult to perform these tests and lead to inaccurate results. 12

In our study, we introduced two recently developed clinical tests, the lever sign and FAB-sign, which have several advantages. Their simplicity, ease of administration, and independence from the examiner's experience make them particularly valuable in challenging clinical scenarios. The results showed that the FAB-sign test has a high specificity in diagnosis, at 97.2%, similar to the Lever sign (94.4%) and Pivot-shift (94.4%). On the other hand, the FAB-sign test has a higher sensitivity (81.4%) compared with the pivot-shift, at 65.9%.

Both the FAB-sign and Pivot-shift tests share a common mechanism for detecting ACL injuries, which is tibial subluxation relative to the femoral condyle. However, the FAB-sign is less painful for the patient, making it potentially easier to elicit a positive response during the examination. The FAB-sign test, developed by Blanke et al., 5 reported a sensitivity and specificity of 78 and 95%. The differences in results compared with our study may be attributed to the original study only including patients with a time period of 6 weeks or more after the injury, 5 while we did not apply a time limitation.

Among the tests, the Lever sign is the one with highest sensitivity and specificity (93.8% and 94.4%, respectively). The authors of the Lever sign test, Lelli et al., 6 reported 100% sensitivity. However, Kulwin et al. 13 showed the results for the sensitivity and specificity of the lever sign test were quite low, at 44.4% and 88.3%, when compared with MRI.

In the meta-analysis studies, the lever sign test has shown relatively favorable results. Huang et al. 9 reported its sensitivity and specificity to be of 79% and 92%, while in a study by Sokal et al., 10 the figures were 83% and 91%, respectively.

In clinical practice, we found that the examination results are influenced by the examination table. If the patient is placed on a padded examination table, it may lead to a false negative result. Therefore, we examine patients on a hard examination table or place a board under the patient's foot, to achieve more accurate results. Additionally, the choice of hand placement under the calf is also crucial in the examination. Our experience is to examine the healthy leg first, select a point under the calf, where lifting the heel results in the highest point, and then use it as a reference to examine the injured leg.

Another factor that can affect the results of the lever sign test is the presence of chondral lesions. Massey et al. 14 observed that its accuracy decreases from 89 to 74% when there is a chondral lesion, with statistical significance. Therefore, this may explain why some studies report a relatively low sensitivity of the lever sign test.

## Conclusion

There are various clinical diagnostic tests for diagnosing ACL tears. An ideal diagnostic method should be easy to perform, highly sensitive, and specific. From this perspective, the lever sign is a useful clinical diagnostic test for physicians to examine and diagnose this condition. Our findings demonstrate that it is not only a practical and simple test but also exhibits superior diagnostic accuracy compared with traditional methods. This makes it an invaluable tool, especially in settings where access to advanced imaging is limited.
